# Microstructure, Properties, and Numerical Simulation of Semi-Solid Aluminum Alloy under Planetary Stirring Process

**DOI:** 10.3390/ma15093009

**Published:** 2022-04-21

**Authors:** Bing Zhou, Zhiyan Qiu, Keping Chen, Chun Xu, Zhanyong Wang

**Affiliations:** School of Materials Science and Engineering, Shanghai Institute of Technology, Shanghai 201418, China; qiujanice@163.com (Z.Q.); m16605162069@163.com (K.C.); zhanyong@sina.com (Z.W.)

**Keywords:** planetary stirring, semi-solid, numerical simulation, microstructure, properties

## Abstract

In order to solve the problem of insufficient convective heat transfer of uniaxial stirred melt, the temperature field and shear rate of melt under planetary stirring were studied based on CFD simulation. The microstructure and properties of this technology were also experimentally studied. The results show that compared with the uniaxial stirring semi-solid technology, the convective heat transfer ability of aluminum alloy, semi-solid slurry in planetary stirring mode is stronger. In addition, its temperature field can be reduced to the semi-solid range faster and more evenly, which is conducive to a large number of nucleation and improves the nucleation rate. The temperature difference of the whole melt is small, so the preferred direction growth and uniform growth of dendrites are avoided, and the morphology is improved. Properly increasing the revolution and rotation speed of the stirring shaft can refine the grains of semi-solid aluminum alloy parts, improve the grain morphology, and improve the tensile strength. The planetary stirring semi-solid process is very suitable for rheological high-pressure casting.

## 1. Introduction

Semi-solid metal forming is a kind of metal forming based on the good rheological properties of the metal in the semi-solid region during the transition of metal from solid to liquid or from liquid to solid. It has the advantages of stable filling, no turbulence and spatter, low processing temperature, and small solidification shrinkage, so the dimension accuracy of the casting is high. The preparation methods of semi-solid slurry include the mechanical stirring method [1], electromagnetic stirring method [2,3], ultrasonic vibration method [4], gas stirring method [5,6], and casting method [7,8,9,10]. The mechanical stirring method includes single screw stirring, double screw stirring [11,12], cone barrel rheological forming [13], and forced convection rheological pulping [14].

With the rapid development of computational fluid dynamics (CFD) technology, it is more and more widely used in stirring simulation [15,16,17]. Certain numerical calculation methods must be used to simulate the preparation and filling process of semi-solid metal slurry. According to the different research objects and methods, numerical calculation methods can be divided into the Lagrange method and Euler method; another mesh-less method is under exploration. The VOF (Volume-of-Fluid) method is currently the most popular and is used in well-known computational fluid dynamics programs, such as ANSYS Fluent, Star-CD, Geo MATH, CFX, and Flow-3D [18,19,20]. One of the basic contents of numerical simulation of melt filling and solidification process is to solve the continuity equation and the Navier–Stokes equations based on finite difference or finite element methods. Liquid metal is usually regarded as an incompressible fluid, and its flow process obeys the conservation of mass and momentum. Its mathematical form is continuity equation and momentum equation.

Through the research on the preparation technology of mechanical stirring semi-solid slurry, it is found that a single stirring shaft can fully stir the melt heat exchange in the center. However the melt far from the center has poor stirring and heat exchange capacity due to the influence of inertial laminar flow. There is a large temperature difference, which takes a long time to make the temperature field of the whole melt uniform, especially when preparing large volume semi-solid slurry. The ability of the stirring shaft to drive melt convection is weaker, the middle temperature of the melt is low, the surrounding temperature is high, the temperature difference is more obvious, and the heat exchange efficiency is lower. Therefore, in order to improve the heat exchange and stirring problem between ordinary stirring shaft and melt, a new planetary composite stirring semi-solid technology with superposition revolution and rotation was used to study the temperature field, microstructure, and properties in the preparation of semi-solid slurry by means of experiment and numerical simulation.

## 2. Experimental Method and Numerical Simulation

A356 aluminum alloy was used as the research material with the solidus and liquidus temperatures of 560 °C and 615 °C, respectively. The alloy was placed in the heating furnace and heated to 710 °C for melting. After slag removal, refining, and other operations, the melt was kept at 660 °C. An appropriate amount of alloy liquid was treated with self-made planetary stirring semi-solid equipment to prepare the aluminum alloy, semi-solid slurry.

**Figure 1:**
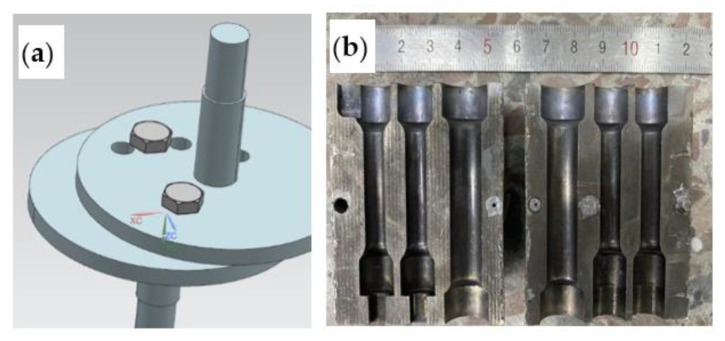
(**a**) Eccentric part of the planetary stirring structure and (**b**) the drawing of tensile sample mold.

The planetary mixing semi-solid equipment includes two independent motion controls of the rotation and revolution of the mixing shaft. The rotation of the mixing shaft is realized by the motor and transmission gear located on an intermediate platform. The revolution of the mixing shaft is realized by the rotation of the motor and eccentric shaft through the intermediate platform. The equipment mainly controls the rotation (300, 500 r/min), rotation (0.7, 1.4 rad/s), and mixing time (15~30 s) parameters to prepare the semi-solid slurry. The whole procedure can be automatically controlled by PLC of Siemens S7-200 SMART (Siemens, Beijing, China). The stirring shaft was put into the melt for a certain time, the melt temperature was monitored in real-time through the thermocouple, and the semi-solid slurry was poured into the die-casting mold or water-cooled spindle mold for solidification. Figure 1 shows the eccentric part of the planetary stirring structure and the drawing of the tensile sample mold.

After rough grinding, fine grinding, and polishing, the microstructure of the sample was observed and photographed by a metallographic microscope of ZEISS Axio Scop A1 (ZEISS, Jena, Germany). The grain size and roundness were analyzed by image processing software (ImageTool, Version2.0 made by Texas health Science Center in San Antonio, TX, USA). The grain size is represented by the average diameter *D* and the roundness is represented by the shape factor *F*. The closer *F* is to 1, the rounder the solid grain is. The specific formula is: $D=\sqrt{\frac{4A}{\pi }}$, $F=\frac{4\pi A}{P^{2}}$. Where *A* is the grain area and *P* is the grain section perimeter. The tensile strength and elongation of the samples were measured by an electronic universal testing of MTS-SANS machine (Ningbo, China).

In order to better understand the effects of mixing action plus revolution action on melt flow behavior and temperature field, the numerical simulation of the semi-solid slurry preparation process was realized. The CFD simulation procedures of the melt stirring by the planetary stirring device are as follows: First, the 3D solid model with actual size is created. Second, the standard joint form IGES file is output. Third, the above model is transmitted to Meshing–Geometry Block. Then, the attributes of material, heat exchange coefficient on boundary, rotational speed, initial temperature, and other Pre-Processing parameters are set. After the calculation, simulation results are directly observed through the Post-Processing block.

**Figure 2:**
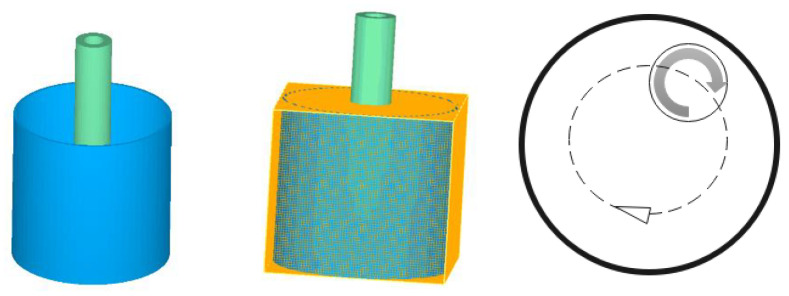
Three-dimensional model, meshing, and mixing operation diagram for simulation.

The effect of rotational condition on the flow characteristics and temperature field of the melt planetary stirring device is important to understanding the nucleation and grain growth mechanism. Figure 2 shows the three-dimensional model, meshing, and mixing operation diagram for numerical simulation. The finite difference method was used to generate the hexahedral mesh of the simplified model. To reduce computing time, the total number of cells is about 360,000 and the width of the grid is 2.2 mm, which has no significant difference from 800,000 cells in the final simulation results.

The fluid material database of simulation software has provided most material thermal properties of the A356 aluminum alloy, such as specific heat, thermal conductivity, latent heat of fluid, and liquidus temperature. The material data can be directly used for simulation with only minor modifications. The viscosity of melt adopts the original fixed value in the database, which should be better if the non-Newtonian fluid model is adopted. However, the current simulation has not been involved. Several important parameters, such as melt temperature, barrel temperature, and rotation condition of the planetary stirring process, which have a significant influence on microstructure, were set in actual conditions. The actual agitating shaft rotation speed has been treated by the reducer, according to the actual situation. The rotational speed is set to 18.84, 31.4 rad/s, and the revolution speed is 0.7, 1.4 rad/s by the moving module. The specific parameters and computation conditions used in the simulation are shown in Table 1.

## 3. Results

### 3.1. Simulation Results

#### 3.1.1. Melt Flow Characteristics of Planetary Mixing and Stirring Process

The research of flow characteristics in the planetary mixing semi-solid device will be helpful to understand the way of heat transfer and the change of temperature, and thus provides a theoretical basis for adjusting process parameters and for controlling solidification process.

**Figure 3:**
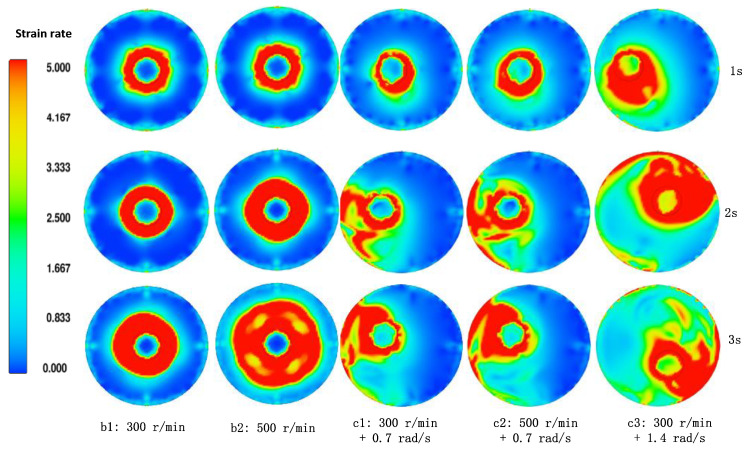
Melt strain rate of planetary mixing and stirring process.

The melt in the planetary mixing semi-solid device has complex stirring-mixed flow characteristics. Figure 3 shows the effect of different revolution and rotation on the shear rate of the melt. It can be seen from Figure 3(b1,b2) that rotation mainly produces a large shear rate on the melt near the stirring shaft, the melt away from the stirring shaft is weakly sheared, and the influence range of rotation on the melt becomes larger with the increase of stirring speed. In Figure 3(c1,c2), the stirring shaft rotates around the middle part of the crucible at a small speed, and the melt on the side of the stirring shaft close to the crucible will be fully sheared and stirred. The melt on the side of the stirring shaft away from the crucible will be sheared weakly and increasing the rotation speed of the stirring shaft cannot change this situation. When the revolution speed of the stirring shaft is increased in Figure 3(c3), the stirring shaft can rotate around the crucible faster, and the melt on the side of the stirring shaft away from the crucible also has a better shear rate. Under the condition of planetary stirring, properly increasing the revolution speed has better shear stirring effect on the whole melt than increasing the rotation speed, but the revolution speed should not be too high to avoid serious turbulent entrainment.

#### 3.1.2. Variation of Melt Temperature Field in Planetary Mixing Stirring Process

**Figure 4:**
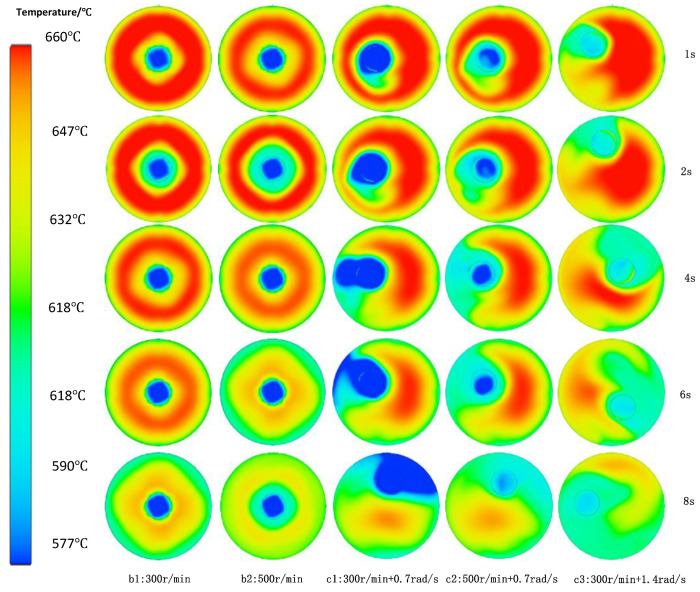
Variation of melt temperature field in planetary mixing stirring process.

The melt has sufficient convection in three dimensions, and the temperature field of the melt in the device will be affected enormously. As shown in Figure 4(b1,b2), under the condition of simple rotation, the melt close to the core of the stirring shaft first exchanges heat with the stirring shaft to cool down. With the increase of stirring speed, the melt cooling speed accelerates. With the extension of stirring time, although the total cooling degree of the two is not much different, it is clearly appropriate to increase the stirring speed, and the temperature field changes faster and more uniform. Under the condition of rotation combined with slow revolution in Figure 4(c1), due to the dual cooling of the mixing shaft and the cylinder at the beginning, the cooling speed of the melt between the mixing shaft and thae cylinder exceeds the degree of simple rotation, which can obtain a very large undercooling in a short time. This occurs while the melt far away from the mixing shaft and the cylinder cools slowly, and there is a very large temperature difference between them. With the increase in rotation speed, the shear flow of melt near the stirring shaft is driven, and the temperature difference between different positions is improved, as shown in Figure 4(c3). When the revolution speed is increased, the whole melt can be driven to high-efficiency heat exchange in a short time, the melt can quickly reach a uniform supercooling state, and the temperature difference of the melt is reduced to the greatest extent, which is conducive to the faster and uniform nucleation and uniform growth of the whole melt. This occurs especially to reduce the hanging of material on the mixing shaft due to the formation of the solidified shell near the mixing shaft due to local supercooling. This is very important for the stable and continuous preparation of semi-solid slurry.

#### 3.1.3. Variation of Melt Temperature Difference with Time under Planetary Stirring Process

**Figure 5:**
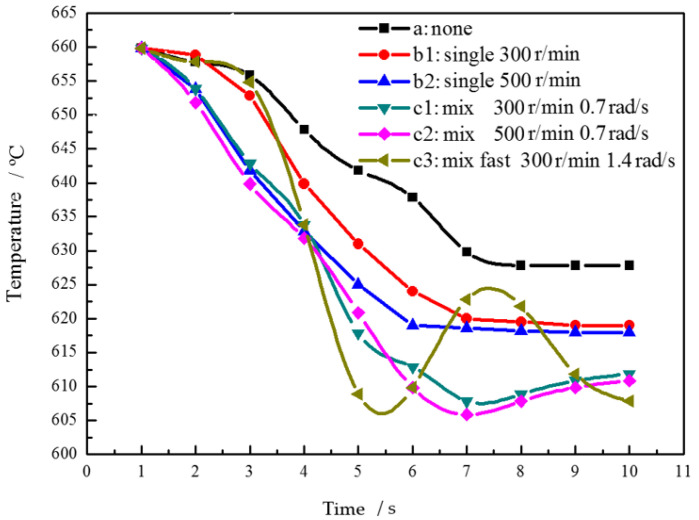
Temperature variation curve of melt with time under different stirring conditions.

When the stirring shaft rotates only in the middle of the melt, the melt heat transfer in the middle of the stirring shaft and the wall lag behind. Therefore, we analyze the melt temperature in this region and the maximum temperature difference of the whole melt. Figure 5 is the curve of melt temperature in the middle of the stirring shaft and vessel wall with different stirring process parameters with time under simulated conditions. Curve A is the temperature change curve of the stirring shaft inserted into the alloy melt without stirring. The melt temperature at the middle position cools slowly. After 8–10 s, its temperature remains at 628 °C and above the liquidus. When the stirring shaft rotates, the melt cooling speed is faster than that without stirring. After 7 s, the temperature stabilizes at a lower 619 °C. When the rotating stirring speed is increased, the melt cooling speed is further accelerated. The hybrid planetary stirring is an efficient stirring method. Even if the slow revolution is superimposed, the melt at the middle position can be stirred and convected to bring greater cooling capacity. The melt in the middle position reaches a lower temperature of 608 °C at 7 s. Under the condition of increasing the revolution speed, the huge temperature fluctuation at the middle melt during the heat exchange process is observed in Figure 5(c3). It shows that the planetary stirring is efficient and the heat transfer is sufficient, which is more conducive to obtain a uniform temperature field, which is unfavorable to dendrite production.

**Figure 6:**
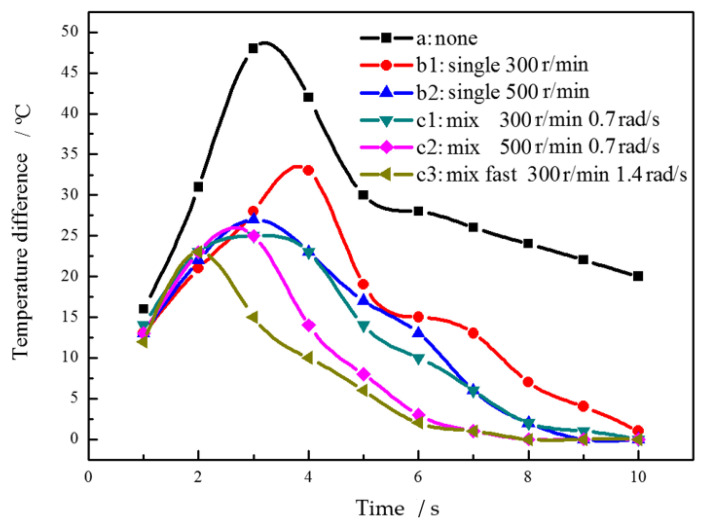
Curve of simulated temperature difference of molten alloy with time.

Figure 6 is the time variation curve of the maximum temperature difference of the whole melt under simulated different stirring process conditions, in order to characterize the temperature variation process of alloy solution. The temperature difference increases first and then decreases with time under six different process parameters. The temperature difference of the alloy solution increases fastest in time without stirring, and the maximum temperature difference can reach 50 °C. Compared with uniaxial slow and fast rotation, the latter is slower than the former in the heating stage, and both of them are slower than that without stirring. In the cooling stage, the decline rate of the latter is slightly higher than that of the former. The maximum temperature difference decreases with the increase of rotation and revolution speed, and the change trend of cooling speed is the same. Under the same conditions, it takes the shortest time for the melt to reach a lower temperature difference under the condition of the rapid revolution and compound. In contrast, slightly increasing the revolution speed is more effective in reducing the maximum temperature difference of the whole melt than increasing the rotation speed.

### 3.2. Microstructure and Mechanical Properties

#### 3.2.1. Microstructure of A356 Aluminum Alloy under Different Stirring Conditions

**Figure 7:**
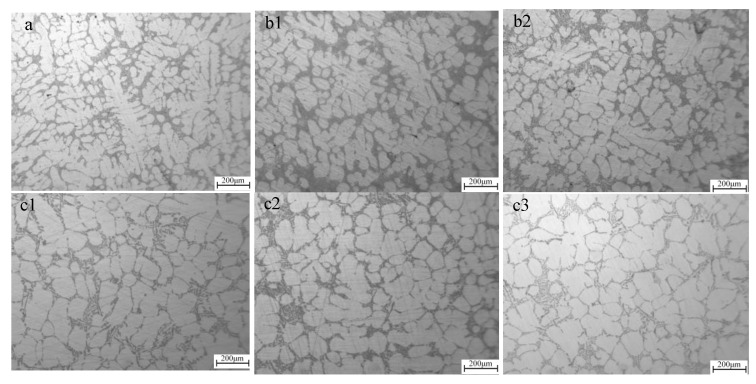
Semi-solid microstructure of A356 alloy under different stirring conditions: (**a**) No stirring; (**b1**) rotation 300 r/min; (**b2**): rotation 500 r/min; (**c1**): rotation 300 r/min revolution 0.7 rad/s; (**c2**): rotation 500 r/min revolution 0.7 rad/s; (**c3**): rotation 300 r/min revolution 1.4 rad/s.

Figure 7 shows the comparison of micromorphology of A356 aluminum alloy semi-solid under different stirring processes. Figure 7a shows the microstructure of A356 aluminum alloy ordinary casting. It can be seen that the structure is a large dendritic structure, which is disorderly and unevenly distributed. Figure 7(b1) shows the semi-solid structure of aluminum alloy prepared by traditional uniaxial rotation. It can be observed that the dendritic structure begins to break and the rose-like grains increase instead. The number of nearly spherical grains in the semi-solid slurry structure obtained in Figure 7(b2) began to increase, but the rosette grains decreased. In Figure 7(c1), the size of the primary solid phase decreases and the number of near spherulites increases. In Figure 7(c2), the grain contour becomes more obvious, and the proportion of spherical grains increases significantly. In Figure 7(c3), the grain shape tends to be round, while the grain size tends to be uniform.

The grain size of A356 semi-solid as cast is about 168 μm. At the same time, the shape factor is 0.46. After the semi-solid was prepared by uniaxial slow rotation and rapid rotation, the grain size gradually decreased to 126 μm. At the same time, the shape factor gradually increased to 0.72. After the semi-solid was prepared by using Figure 7(c1–c3) composite rotation and revolution methods. The grain size gradually decreased to 119 μm again. At the same time, the shape factor gradually increased to 0.8 again.

#### 3.2.2. Mechanical Properties of A356 Semi-Solid under Different Stirring Processes

**Figure 8:**
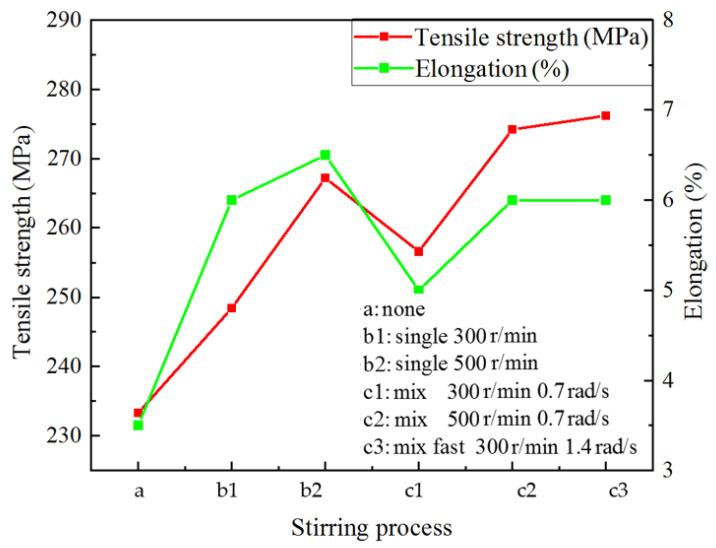
Mechanical properties of A356 semi-solid alloy under different stirring processes.

Figure 8 shows the mechanical properties of A356 semi-solid under different stirring processes. Under the condition of uniaxial rotation, increasing the stirring speed from 300 r/min to 500 r/min increased the tensile strength by 18.8 MPa and the elongation by 2.5%. The performance is improved by 7 MPa when a small revolution speed is superimposed compared with the single shaft rotation of 300 r/min. When the rotating speed of the mixing shaft is 500 r/min, the composite mixing is 8.2 MPa higher than that of single shaft rotation. Under the condition of compound rotation and revolution, increasing the rotation speed increased the tensile strength by 17.6 MPa and the elongation by 1%. Increasing the revolution speed, the tensile strength of the test bar was increased by 19.9 MPa. It should be noted that the elongation at stirring speed of 300 r/min + 0.7 rad/s is lower than that at 300 r/min alone. The possible reason is that the stirring shaft is too close to the crucible wall, and the slurry between the crucible wall and the stirring shaft cools faster than that during central stirring, while too slow revolution speed does not have much effect on the uniform heat transfer of the melt, as shown in Figure 4c. There may also be a reaction, resulting in a reduction in elongation. However, with the increase of stirring speed, this problem has been improved. Therefore, under planetary stirring conditions, appropriately increasing the stirring shaft speed or increasing the revolution speed can improve the tensile properties of aluminum alloy, increasing the revolution speed more effectively.

#### 3.2.3. Microstructure of Al-Si Alloy of Planetary Stirring Semi-Solid Die Casting

**Figure 9:**
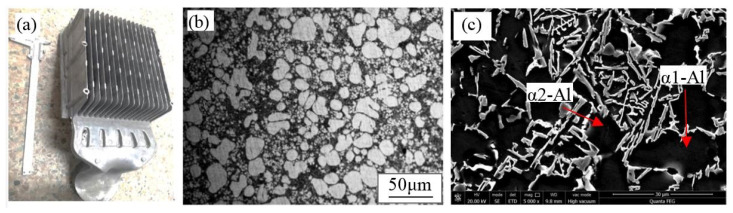
The microstructure of Al-Si alloy communication parts by planetary stirring semi-solid technology. (**a**) Communication cooling shell part, (**b**) Metallographic diagram, (**c**) SEM diagram.

Figure 9 shows the microstructure of Al-Si alloy communication parts cast by planetary stirring semi-solid technology. We combine planetary stirring semi-solid technology with die casting to trial produce semi-solid die castings. Figure 9a is a diagram of actual communication cooling shell part. The parameters of the process are the initial melt temperature of 670 °C, rotational speed of 300 r/min, and revolution speed of 1.4 rad/s. The structure at the position of the heat sink of the part is cut for polishing and corrosion, and then analyzed by metallography and scanning electron microscope. As can be seen from Figure 9b,c, the microstructure of the semi-solid die castings is mainly composed of rosiness and nearly spherical primary α-Al particles, and the non-dendritic primary particles are uniformly dispersed in the liquid matrix. The particles size is about 20 μm, which is smaller than normal solidification. Because the quenching effect of the die cavity on the generation of new particles is obvious with significant increases in the number of the particles. The melt at the blades takes a shorter solidification time due to the thinner thickness and the morphology of particles is relatively rounder than the particles in other areas. In addition to the small and rounded primary grains (α1-Al), the average size of the secondary aluminum phase (α2-Al) of semi-solid slurry is decreased to 5~10 μm and the number is increased significantly.

## 4. Discussion

### 4.1. Analysis of Melt Temperature Field under Planetary Stirring Process

Based on the previous numerical simulation and experimental results, the mechanism between melt temperature change and grain nucleation and growth during composite stirring pulping is analyzed and discussed. Composite stirring superimposes revolution on the basis of traditional uniaxial spin. Compared with traditional uniaxial stirring, composite stirring drives the flow range of melt larger and has higher efficiency.

The faster the rotating speed of the stirring shaft or the faster the revolution speed of the stirring shaft, the faster the cooling of the alloy liquid, and the shorter the time required for the alloy liquid to reach the minimum temperature. The higher the revolution speed of the stirring shaft, the higher the convection intensity of the alloy melt driven by the stirring shaft, and the faster the convective heat exchange between the melt with higher temperature and the melt with lower temperature. In the case (c3 in Figure 4), the temperature in the middle of the alloy liquid changes greatly in a short time, and the temperature decreases first and then increases. The temperature of the alloy liquid appears at a peak.

Under the same conditions, the temperature difference of the alloy liquid without stirring is the largest, which makes the grain growth direction most obvious and easy to form dendrites. Under the traditional single-shaft slow stirring, the stirring shaft drives a small part of the surrounding liquid to rotate, which can reduce the temperature of the alloy liquid near the stirring shaft. The heat of the alloy liquid diffuses slowly outward from the stirring shaft, resulting in a smaller temperature difference between the alloy liquid at the same time, better cooling effect, and less dendritic crystals than that without stirring. The high-speed rotation of the stirring shaft of the traditional uniaxial rapid stirring drives the convection and exchange of the surrounding alloy liquid. Compared with the first two methods, the temperature difference is the smallest at the same time in the falling stage, which is more conducive to the formation of round-shaped grains.

Composite stirring has one revolution more than uniaxial stirring, which expands the volume of alloy liquid involved in the convective exchange and makes the solution reduce to the minimum temperature more quickly. In this way, the overall temperature difference of alloy liquid is also smaller. The directionality of the grain solidification process is smaller. These can reduce the possibility of dendrite formation. The composite fast stirring reduces to the lowest temperature faster than the composite slow stirring, and its convection intensity is stronger than the latter. The decrease of stirring temperature difference with time is the fastest, indicating that the convective exchange between alloy liquids is the most effective.

From the temperature field and temperature difference curves of alloy liquid under several stirring modes, the melt has greater overall undercooling under composite stirring. With the increase of the rotation and revolution speed of the stirring shaft, the lower the overall temperature of the alloy liquid, the smaller the temperature difference and the greater the undercooling. Therefore, heterogeneous nucleation requires smaller nucleation work and critical nucleation radius, which is more conducive to nucleation.

### 4.2. Grain Nucleation and Growth Mechanism under Planetary Stirring

The classical theory of semi-solid, near-spherical grain formation is dendrite fragmentation spheroidization. According to the different action modes of an external field, the mechanisms can be divided into dendrite arm mechanical breaking, fusing, and dendrite arm bending induced grain boundary liquid infiltration [21]. Although there are differences, the main viewpoints are that the near-spherical grains are spheroidized after the dendrite formed in the supercooled solution is broken. In recent years, many scholars have found the direct growth of near-spherical crystals in the melt. Li Tao et al. [22] observed the direct formation process of near-spherical grain structure of Sn-15%Pb alloy during solidification. A. Das and Z. Fan et al. [23] studied the double helix stirring semi-solid technology and believed that the penetration of metal liquid into the dendrite arms increased the growth rate of the root and the side of the primary grain dendrite arms, so as to grow into a rose shape. Pilling [24] analyzed and studied the effect of shear rate on grain dissociation through the stress calculation model of the dendrite wall. Zhou Bing [25] and Zhang Jingxin [26] respectively studied the influence and control factors of a uniform temperature field formed by melt convection caused by mechanical stirring and electromagnetic stirring on nucleation and growth. More and more scholars accept the theory of directly controlling melt nucleation and growth into near-spherical grains under stirring treatment.

Compared with the traditional stirring, the melt is disturbed at a smaller shear rate in the container due to the existence of inertia. Compared with the traditional stirring, the melt is disturbed at a smaller shear rate in the container due to the existence of inertia. Planetary stirring has two behaviors of rotation and revolution, which has more efficient stirring and mixing effect and can avoid laminar flow caused by simple rotation, which is very conducive to the uniformity of the temperature field and promotes the overall nucleation.

**Figure 10:**
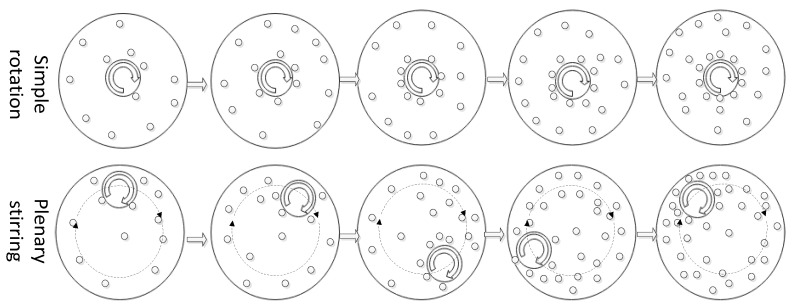
Diagram of crystal dissociation of different stirring process.

According to classical solidification theory, for homogeneous nucleation and heterogeneous nucleation, the critical nucleation radius is inversely proportional to the degree of supercooling. The critical nucleation energy varies inversely as the square of the supercooling degree. Compared with the traditional uniaxial rotation, the melt under the condition of planetary stirring can drop to a lower semi-solid temperature range in a shorter time, reach a lower undercooling faster, reduce the critical nucleation energy and critical nucleation size, and nucleate more easily. At the same time, it will also increase the scouring effect on nucleated grains and increase the number of free grains. The grain dissociation process is shown in Figure 10.

Compared with traditional uniaxial stirring, the melt under composite stirring can achieve a more uniform temperature field in a shorter time, reduce the temperature difference and undercooling gradient of the whole melt, and the spin and collision of grains under stirring conditions will also change their local temperature field environment, avoiding the preferred directional growth of grains to the greatest extent. The semi-solid slurry with a large number of fine, uniform, and round grains is obtained.

Rheological die casting was carried out by combining planetary stirring semi-solid technology with a die casting machine. Compared with superheated melt, semi-solid slurry has more primary grains and lower melt temperature. Lower melt temperature leads to larger supercooling degree and smaller solidification shrinkage. In the subsequent filling and solidification processes inside the mold, the time used to primary grain growth and secondary grain generation is shorter. Due to the large supercooling degree, the secondary nucleation has a great driving force, resulting in a large number of secondary grains. The grain size and morphology of α1-Al/α2-Al in microstructure was significantly refined as shown in Figure 9. Conversely, the semi-solid slurry has unique performance of high viscosity and good fluidity. The high viscosity of the slurry can reduce the turbulence and splash in the filling process. Less entrapped gas content and smaller solidification shrinkage can effectively reduce the porosity of castings. The castings prepared with semi-solid slurry have better comprehensive performance.

## 5. Conclusions

Compared with the common cast dendrite structure, uniaxial rotation improves the microstructure and properties of A356 aluminum alloy, and its average grain size ranges from 145 μm increased to 126 μm. The shape factor is increased from 0.59 to 0.72, the tensile strength is increased from 248 MPa to 267 MPa. Under the condition of planetary stirring, with the increase of stirring speed, the size of the primary solid phase is about 132 μm reduced to 119 μm. The shape factor increased from 0.65 to 0.8, the tensile strength increased from 256 MPa to 276.2 MPa, and the elongation increased to 6%. The semi-solid slurry prepared by planetary stirring is convenient to be combined with die casting, and the grain size of Al-Si alloy is only 20 μm.

The increase of stirring speed is conducive to improve the convective heat transfer of alloy melt, reduce the overall temperature of melt, increase the undercooling, and increase the number of nucleation. Planetary stirring is a complete stirring of the whole melt without a dead angle, which avoids the inertial laminar flow during uniaxial stirring, has higher mixing heat exchange efficiency, can reduce the melt to a greater undercooling faster, and promotes the overall uniform nucleation. At the same time, the convective shear effect of composite stirring is also conducive to grain dissociation and increases the number of nucleation. Conversely, by increasing the nucleation rate, planetary stirring can obtain smaller temperature differences faster, reduce undercooling gradient, avoid preferential growth of melt, and is conducive to uniform growth.

## Figures and Tables

**Table 1:** Process and initial conditions of the simulation.

Input	Value	Unit
Initial melt temperature	660	°C
Initial barrel temperature	560	°C
Initial agitating shaft temperature	25	°C
Agitating shaft rotation speed	18.84, 31.4	rad/s
Agitating shaft revolution speed	0.7, 1.4	rad/s
Thermal conductivity of agitating barrel	23.4	W/m·K
Thermal conductivity of agitating shaft	23.4	W/m·K
Heat transfer to melt	7	kW/m^2^·K
Heat transfer to air	5	kW/m^2^·K

## Data Availability

Not applicable.
